# The mechanical properties of the mantle muscle of European cuttlefish (*Sepia officinalis*)

**DOI:** 10.1242/jeb.244977

**Published:** 2022-12-15

**Authors:** Nicholas W. Gladman, Graham N. Askew

**Affiliations:** School of Biomedical Sciences, Faculty of Biological Sciences, University of Leeds, Leeds, West Yorkshire LS2 9JT, UK

**Keywords:** Cephalopod, Muscle mechanics, Ontogeny, Power output

## Abstract

The circular muscles surrounding the mantle cavity of European cuttlefish (*Sepia officinalis*) generate the mechanical power to compress the cavity, forcing a jet of water out of the funnel, propelling the animal during jet propulsion swimming. During ontogeny, jetting frequency decreases in adults compared with juveniles, and this is expected to be reflected in the contractile properties of the locomotory muscles. To develop greater insight into how the locomotion of these animals is powered during ontogeny, we determined the mechanical properties of bundles of muscle fascicles during isometric, isotonic and cyclic length changes *in vitro*, at two life stages: juveniles and adults. The twitch kinetics were faster in juveniles than in adults (twitch rise time 257 ms compared with 371 ms; half-twitch relaxation 257 ms compared with 677 ms in juveniles and adults, respectively); however, twitch and tetanic stress, the maximum velocity of shortening and curvature of the force–velocity relationship did not differ. Under cyclic conditions, net power exhibited an inverted U-shaped relationship with cycle frequency in both juveniles and adults; the frequency at which maximum net power was achieved was shifted to lower cycle frequencies with increased maturity, which is consistent with the slower contraction and relaxation kinetics in adults compared with juveniles. The cycle frequency at which peak power was achieved during cyclical contractions *in vitro* was found to match that seen *in vivo* in juveniles, suggesting power is being maximised during jet propulsion swimming.

## Teaser

**Summary:** Slowing of muscle contraction kinetics during development underlies the shift in the optimal frequency for maximal power output during jet propulsion swimming in cuttlefish.

## INTRODUCTION

Jet propulsion swimming is a key part of cephalopod locomotion, playing key roles in escape and predation, as well as routine swimming (Bartol et al., 2001; Wells, 1995). The production of jets is powered by muscles in the mantle, which compress and increase the pressure of the water in the cavity, resulting in its expulsion via the siphon and the generation of thrust (Bartol et al., 2001; Kier and Schachat, 2008; Milligan et al., 1997). The mantle muscle of squid and cuttlefish contains two types of obliquely striated muscle: the radial muscles, which act to expand the mantle cavity, facilitating water intake, and the circular muscles, which act to compress the mantle cavity (Kier and Schachat, 2008; Kier and Thompson, 2003). The circular muscles contain two distinct layers, termed the ‘central mitochondria poor’ and the ‘superficial mitochondria rich’ musculature (Shaffer and Kier, 2012; Thompson and Kier, 2006), which are analogous to the fast- and slow-twitch fibres of vertebrates, respectively. These different muscle types perform distinct functions: the superficial mitochondrial rich fibres are involved in routine swimming and respiration; the central mitochondria poor fibres are involved in escape responses (Bartol et al., 2001; Kier and Schachat, 2008; Kier and Thompson, 2003).

Characterisation of the contractile properties of cephalopod muscle has been limited to measurements during isometric and isotonic contractions, in squid (longfin squid, *Doryteuthis pealeii*: Thompson et al., 2010, 2008; European common squid, *Alloteuthis subulata*: Milligan et al., 1997; Rogers et al., 1997), and European common cuttlefish (*Sepia officinalis*: Curtin et al., 2000; Milligan et al., 1997; Rogers et al., 1997), and has mostly focused upon mature animals (but see Thompson et al., 2010; Lamarre et al., 2019), with little work addressing whether changes occur throughout ontogeny (Thompson et al., 2010). However, during jet propulsion swimming, the mantle muscle contracts intermittently, undergoing a cyclical length change during which power is generated, which is rather different to muscle behaviour during isometric and isotonic contractions (Caiozzo, 2002; Josephson, 1993). The aim of this study was to determine the contractile properties of the mantle muscle during cyclical contractions, to obtain a better understanding of how these muscles power jet propulsion swimming. We hypothesised that isometric twitch rise and relaxation times would increase in adults compared with juveniles, as has been noted during ontogeny in vertebrates (Altringham et al., 1996; Johnson et al., 1993; Van Wassenbergh et al., 2007). Alongside changes in isometric properties, we hypothesised the power–frequency relationship would shift to lower cycle frequencies with increased animal size, and that mechanical power would be highest at cycle frequencies used during escape swimming.

## MATERIALS AND METHODS

### Animals

Mature European cuttlefish (*Sepia officinalis*, Linnaeus 1758) were captured in Poole harbour in April 2016 (JHC research, Poole, Dorset, UK). Cuttlefish were reared from eggs taken as by-catch upon fishing gear in the English Channel (RK Stride, Christchurch, Dorset, UK) during May 2016.

Juveniles were reared from eggs and housed in 500, 360 and 300 l (length×width×height: 1300×800×460 mm, 910×690×570 mm and 890×590×550 mm) recirculating artificial saltwater systems at the University of Leeds. Water temperature and salinity were maintained at 19±1°C and 32±1 PSU {formulated using Aqua One Reef synthetic [Kong's (Aust.) Pty Ltd, Sydney, NSW, Australia] in deionised water} to maximise development speed (Bouchaud, 1991). Once eggs had begun hatching, the temperature of the water was gradually decreased to 15±1°C over a period of 10 days. Animals were fed twice daily using size-appropriate live foods: enriched *Artemia salina* (Vitalis live food enrichment, World Feeds Ltd, Thorne, Derbyshire, UK; Peregrine Livefoods, Magdalen Laver, Essex, UK), *Mysis* shrimp (*Mysis* spp.; Aquadip VOF, Oss, North Brabant, The Netherlands; Essex Marine Aquatics, Wickford, Essex, UK), and river shrimp (*Palaemon varians*; Aquatic Live fish foods, Woodford, London, UK). Cuttlefish were reared until a mantle length of 30–45 mm was reached in groups of 100 animals.

Mature animals were maintained in 500, 360 and 300 l (length×width×height: 1300×800×460 mm, 910×690×570 mm and 890×590×550 mm) recirculating artificial saltwater systems at the University of Leeds in size-matched groups of 3–5 animals. Water was maintained at a temperature and salinity of 11±1°C and 32±1 PSU, matching the conditions at the site from which animals were taken during April 2016. Animals were fed twice daily using langoustine (*Nephrops norvegicus*), tiger prawns (*Penaeus monodon*) or live shore crab (*Carcinus maenas*; Tacklebox mail order, Worthing, West Sussex, UK).

Temperature and salinity were monitored twice daily to ensure that they fell within the natural range of animals in the English Channel at the time eggs or animals were collected (Cefas 2012 data: https://www.cefas.co.uk/data-and-publications/sea-temperature-and-salinity-trends/). pH and nitrogenous compounds were monitored monthly to ensure that animal health and welfare would not be compromised; recommended levels were maintained by carrying out 25% water changes twice per week for tanks housing juveniles, and daily for adult tanks.

### Muscle preparation

Cuttlefish were euthanised in accordance with Schedule 1 of the Animals (Scientific Procedures) Act 1986 (amended 2012); animals were anaesthetised in 3.5% magnesium chloride hexahydrate (VWR Chemicals, Radnor, PN, USA; Flurochem Ltd, Hadfield, Derbyshire, UK) prior to destruction of neuronal tissues (the brain, vertical and optical lobes) via pithing.

A bundle of muscle fibres was dissected from the central zone of the ventral mantle, approximately 40% of the mantle length (measured from the tip of the ventral mantle). Muscle dissections were carried out by hand with the mantle submerged in chilled (11°C) artificial seawater, and pared down to include only the central zone of the muscle. Suture thread (2-0 USP, black braided silk non-absorbable, non-sterile surgical suture, LOOK surgical specialities corporation, Reading, PA, USA) was tied around each end of the muscle preparation and used to attach a stainless-steel ring to each end of the preparation. The muscle preparation was placed in a flow-through Perspex^®^ chamber continually re-circulated with artificial seawater at 11±0.5°C saturated with 100% oxygen. One end of the muscle was attached to a fixed mount and the other to the arm of an ergometer via the stainless-steel rings (Aurora Scientific Dual-mode lever system model 300B-LR, Aurora Scientific Inc., Aurora, ON, Canada). Following dissection, the muscle preparation (mass 80±10 mg) was held at approximately resting length (13±0.40 mm) and left for 1 h to recover (following Olson and Marsh, 1993).

### Isometric contractile properties

The length of the muscle preparation was optimised to yield maximum active twitch force via a series of isometric twitches at different lengths (see Fig. S1 for example force–length profiles), varying by 0.5 mm (measured using callipers and adjusted using a micromanipulator), with stimulation delivered via parallel platinum wire electrodes that ran along the full length of the muscle. The length at which peak twitch force was recorded was defined as *L*_0_. The muscle was allowed to rest for 3 min between each successive twitch contraction. Peak twitch force (*P*_tw_), the time to peak twitch force (*t_P_*_tw_), and time to half-twitch relaxation (RT_50_; time from *t_P_*_tw_ to 50% *t_P_*_tw_) were determined from the isometric twitch which yielded the highest force. An isometric tetanic contraction was generated using a 500 ms train of stimuli (pulse width 0.2 ms; stimulation frequency of 50 Hz).

### Isotonic contractile properties

The force–velocity relationship was determined using a series of after-loaded isotonic contractions at a stimulation frequency of 50 Hz. A control isometric tetanus was carried out after every three isotonic contractions, and a 5 min rest period was allowed between each contraction. Where there was a decline in the control isometric tetanic force, a linear decline was assumed, and this was used to estimate peak isometric force (*P*_0_) at the time each contraction was performed and hence used to determine the relative force (*P*/*P*_0_) for each of the shortening contractions. A hyperbolic-linear curve (Marsh and Bennett, 1986) was fitted to a plot of shortening velocity against relative force to determine the force–velocity relationship (using IgorPro version 7.00, WaveMetrics Inc., Portland, OR, USA) and the maximum shortening velocity (*V*_max_), power ratio (a measure of the curvature of the force–velocity relationship, calculated as Π_i_/*V*_max_*P*_0_; following Marsh and Bennett, 1986) and maximal isotonic power (Π_i_) were determined.

### *In vivo* estimation of muscle strain

Changes in muscle length *in vivo* during jet propulsion escape swimming were estimated using a modification of the methods of Girgenrath and Marsh (1997). The changes in the diameter of the mantle of juvenile cuttlefish were recorded using a Photron FASTCAM SA3 (Photron USA, San Diego, CA, USA) high-speed camera (1024×1024 pixels, 500 frames s^−1^; shuttered at ^1^/_500_ frames s^−1^) mounted perpendicular to the experimental tank (length×width×height: 610×460×450 mm; volume: 126 l) as animals swam through the tank. Sequences were analysed using ImageJ 1.48 (Schneider et al., 2012). The diameter of the mantle cavity was determined prior to a jet event starting, and at the end of the jet exhaust phase. The contraction phase was defined as the period over which a jet was produced, while the refill phase was assumed to be the time between two sequential jet events. The mantle circumference was estimated by assuming that the mantle cavity is circular, and strain was calculated by presenting the circumference relative to the maximum value recorded. We note this method only gives an estimate of the strain of the superficial fibres, and does not account for differences in strain which may occur as a result of the position in the mantle wall (as previously noted by Thompson et al., 2014, in longfin squid).

### Cyclic contractile properties

To determine the net power generated during cyclical contractions which simulate the muscle's performance during jet propulsion swimming, the muscle preparation was subjected to five cycles of sinusoidal length changes and phasic stimulation, using the work loop technique (Josephson, 1985). This waveform closely matches changes in the diameter of the mantle cavity during free swimming (Bartol et al., 2001). The cycle frequencies ranged from 0.6 to 2 Hz (see Table S1) and a strain amplitude of ±0.075 *L*_0_ was used for all cycle frequencies (total strain of 0.15 *L*_0_). This strain amplitude was found to yield maximal net work output during preliminary measurements at a cycle frequency of 1 Hz. The timing and duration of stimulation were optimised to yield the maximum net work, at a stimulation frequency of 50 Hz (pulse width 0.2 ms). The optimum phase of stimulation was found to be invariant and was kept constant at 100 ms prior to peak length. Control work loops at a cycle frequency of 1 Hz were performed periodically to monitor and allow correction for decline in the preparation. In correcting for any decrease in performance, a linear decline was assumed between the control contractions. A decline in control power of up to 20% was noted; however, control power could generally be recovered by resting preparations for 1 h. The net mechanical power and net work generated were determined as the average values from cycles 3 and 4.

At the end of the experiment, the muscle preparation was removed, the sutured ends of the muscle were removed, excess saline was blotted from the muscle and the mass was recorded. This mass was used in the normalisation of force to calculate muscle stress and muscle mass-specific work and power. In determining muscle stress, the density of the muscle was assumed to be 1060 kg m^–3^ (Mendez and Keys, 1960). The radial muscle fibres (running perpendicular to the circular fibres) were still present in the preparations, but did not contribute to the force generated. The radial fibres represent a minor proportion of the total mass of the preparation, estimated to be up to 2.5% of the total mass of the preparation (Ward and Wainwright, 1972). In calculating muscle stress, we ignored the mass of the radial fibres.

### Statistical analysis

Data were analysed using Igor Pro (version 7.00, WaveMetrics Inc., Portland, OR, USA), and IBM SPSS Statistics 24 (International Business Machines Corporation, Armonk, NY, USA). Data are presented as means±s.e.m. All data were tested for normality and homogeneity prior to statistical testing; if data were non-normally distributed, they were transformed to meet these assumptions via log or arcsine transformation. A critical *P*-value of 0.05 was used to indicate significant differences. Where simple comparisons were made, *t*-tests were used. ANOVA tests with Tukey *post hoc* tests were used to test for differences in net work or net power output of muscle between cycle frequencies.

## RESULTS

### Muscle contractile properties

There was a 3.7-fold increase in mantle length between the juvenile and adult stages (juvenile: 45.4±1.1 mm, *n*=5; adult: 166.9±23.9 mm, *n*=8; *t=*14.46, d.f.=7, *P*<0.001). A summary of muscle contractile performance is presented in Table 1, alongside published data from other species of mollusc, for comparison.

**Table 1.**
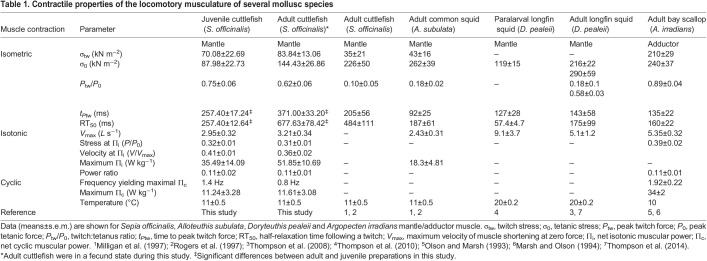
Contractile properties of the locomotory musculature of several mollusc species

### Isometric contractile properties

The twitch (σ_tw_) and tetanic (σ_0_) stresses and the twitch:tetanus ratio did not differ significantly between juvenile and adult animals (σ_tw_: *t=*−0.16, d.f.=11, *P*=0.88; σ_0_: *t=*1.60, d.f.=11, *P*=0.14; *P*_tw_:*P*_0_: *t=*−1.49, d.f.=11, *P*=0.16; Table 1). Isometric twitch stress was 17% higher in adults; isometric tetanic stress was 39% higher in adults (Table 1). Twitch rise and half-relaxation times were significantly slower in adults than in juveniles (*t_P_*_tw_: *t=*3.04, d.f.=11, *P*=0.01; RT_50_: *t=*5.29, d.f.=11, *P*<0.001): isometric twitch rise time was 31% slower in adults and time to twitch half-relaxation was 62% slower in adults (Table 1). During the isometric tetanic contractions, a plateau in force was not attained, as previously noted in cephalopod mantle muscle (Milligan et al., 1997).

### Isotonic contractile properties

**Fig. 1.**
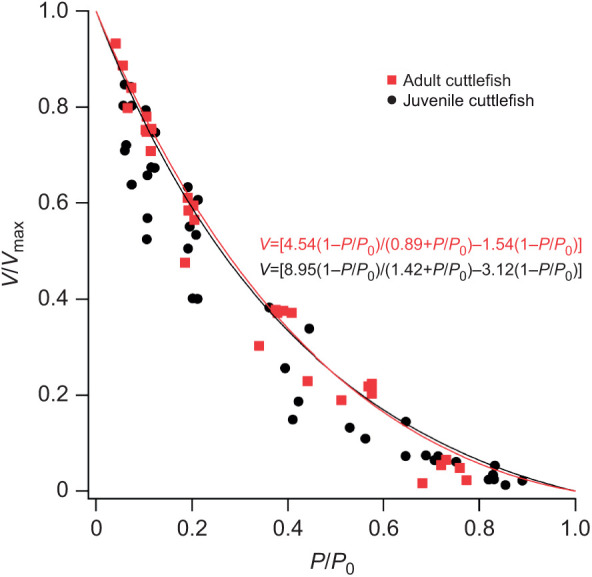
**Isotonic contraction of juvenile and adult cuttlefish muscle preparations.** Mean relationship between relative force (*P*/*P*_0_) and relative velocity (*V*/*V*_max_) of shortening (*n*=5 juveniles, *n*=6 adults). Data were fitted according to the hyperbolic-linear equation: *V*=[*B*(1−*P*/*P*_0_)/(*A*+*P*/*P*_0_)]+C(1−*P*/*P*_0_); the mean equation for each group is displayed.

The maximum velocity of shortening (*V*_max_) was 8% slower in juvenile preparations, with no significant change with age (*t=*0.56, d.f.=10, *P*=0.59; Fig. 1). The maximum isotonic power showed no significant differences between cuttlefish age classes (*t=*0.92, d.f.=10, *P*=0.38; Table 1). The relative force (*P*/*P*_0_) and relative velocity (*V*/*V*_max_) at which maximum isotonic power was attained did not differ significantly between juveniles and adults (*P*/*P*_0_: *t=*−0.74, d.f.=10, *P*=0.48; *V*/*V*_max_: *t=*−0.49, d.f.=10, *P*=0.63; Table 1). The similarity between the relative force and relative velocity at which maximum isotonic power was attained in juvenile and adult muscles indicates that the force–velocity relationships are similar in shape. This is also reflected in the power ratios, which were not significantly different between adults and juveniles (*t=*0.19, d.f.=8, *P*=0.85; Table 1).

### *In vivo* muscle activity

During free-swimming, overall strain (relative to maximum mantle circumference) was 0.19±0.04, ranging from 0.09 to 0.31, which encompassed the strain used for the *in vitro* measurements (strain 0.15; strain amplitude 0.075). The jet contraction lasted 0.44±0.10 s, and mantle refill lasted 0.39±0.07 s, giving a duty cycle of 51.79±8.45% and a cycle frequency of 1.39±0.23 Hz. Assuming that the diameter of the juvenile cuttlefish mantle is proportional to circular muscle strain, strain rate was estimated to be 0.53±0.11 s^−1^.

### Muscle performance during cyclical contractions

**Fig. 2.**
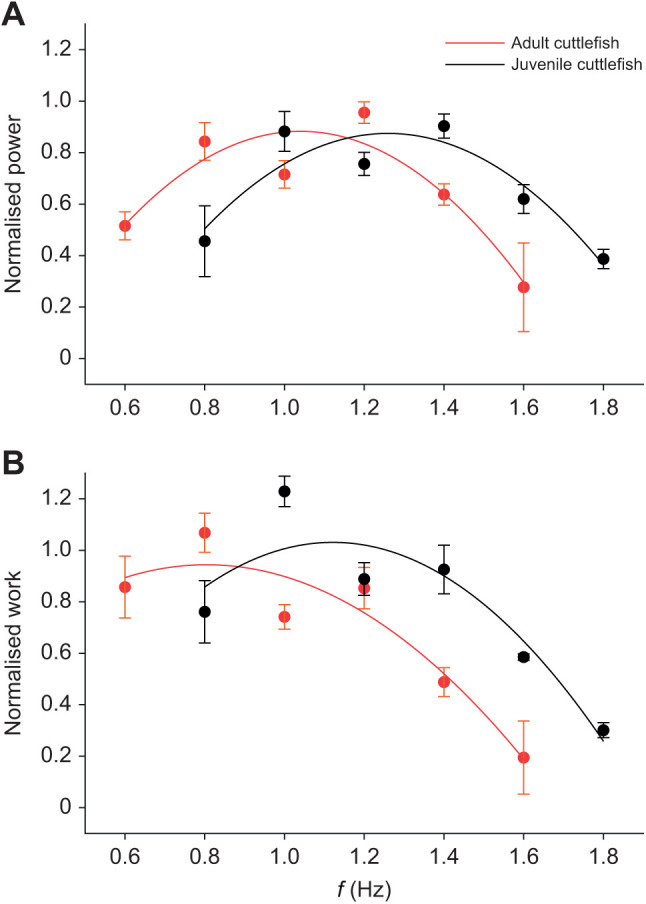
**Relationship between mantle muscle cycle frequency and muscular power and work of juvenile and adult cuttlefish preparations subject to sinusoidal work.** (A) Muscular power normalised to peak power and (B) muscular work normalised to work at the peak power cycle frequency (*f*). Data represent means±s.e.m. (*n*=5 juveniles, *n*=7 adults).

The mean peak net power output was 11.24±3.28 and 11.61±3.08 W kg^−1^ in juveniles and adults, respectively, and was achieved at a cycle frequency of 1.4 Hz (juveniles) and 0.8 Hz (adults) (Table 1, Fig. 2). At cycle frequencies above and below the optimum for maximum power generation, net power decreased in both juveniles and adults. This inverted U-shaped relationship was even more pronounced when power was normalised to maximum net power for each muscle preparation (Fig. 2A). Net power did not differ significantly across the cycle frequency range studied in juvenile (*F*=0.61, d.f.=6, *P*=0.72) and adult muscle preparations (*F*=0.88, d.f.=5, *P*=0.51). The mean peak net work was generated at a cycle frequency of 1 Hz in juvenile preparations and 0.8 Hz in adult preparations, with net work of 9.30±1.91 and 14.51±3.85 J kg^−1^, respectively. Net work did not vary with cycle frequency in juvenile (*F*=1.19, d.f.=6, *P*=0.35) or adult muscle preparations (*F*=2.47, d.f.=5, *P*=0.063; Fig. 2). Net work and net power output did not differ significantly between juveniles and adults (*F*=1.47, d.f.=4, *P*=0.23); however, at 0.8 Hz, adults had significantly higher work and power output (*t=*−2.11, d.f.=6, *P*=0.04).

## DISCUSSION

### Contractile characteristics of obliquely striated musculature

Twitch contraction kinetics (time to peak twitch force and half-twitch relaxation) were slower in adult than in juvenile cuttlefish mantle muscle. During ontogeny in other cephalopods (e.g. longfin squid; Table 1; Thompson et al., 2010) and vertebrates, twitch kinetics have also been found to become slower with increasing body size (James et al., 1998; Marsh, 1990; Van Wassenbergh et al., 2007). For example, in paralarval longfin squid, time to peak twitch force and time to half-twitch relaxation were 89% and 37% those in adults, respectively (Thompson et al., 2010). During ontogeny, the cycle frequency at which the muscle operates decreases with increasing body mass, which is reflected in the intrinsic contractile properties of muscles (Marsh, 1988). During cyclical activities, muscles must be sequentially activated and deactivated in order that force is generated phasically. The time to peak twitch force and half-twitch relaxation time are determined by the deactivation of crossbridge cycling (Marsh, 1990). As rapid deactivation is energetically costly, the contractile properties of the muscle are tightly coupled with the operating frequency during movement, with a specific ratio being maintained between contraction kinetics and cycle duration regardless of whether the muscle is activated by a single stimulus or a burst of stimuli (Askew and Marsh, 2001; Marsh, 1990). The twitch kinetics were similar to those previously reported (time to peak twitch force 205 ms, half-twitch relaxation 484 ms; Milligan et al., 1997) in cuttlefish, though the age of animals used in the earlier study is unclear.

The isometric tetanic stress of cuttlefish muscle (88 kN m^−2^ in juveniles and 144 kN m^−2^ in adults) is within the same range as previously reported values for this species (104±18 kN m^−2^ in juveniles: Lamarre et al., 2019; and 226±19 kN m^−2^ in adults: Milligan et al., 1997). Twitch stress (70 and 84 kN m^−2^) and tetanic stress were also similar to those of other species of mollusc; e.g. twitch stress and tetanic stress of sea scallops (*Placopecten magellanicus*) were 77 and 132 kN m^−2^, respectively (Rall, 1981), though higher values have been reported in other scallop species, such as the bay scallop, where twitch stress and tetanic stress were 210 and 240 kN m^−2^ (Marsh and Olson, 1994; Olson and Marsh, 1993). The lower stress in cuttlefish muscle compared with some scallop adductor muscles may be due to the higher proportion of non-contractile elements (such as mitochondria) in cuttlefish muscle (Thompson et al., 2008). Neither twitch nor tetanic isometric stress varied during ontogeny in cuttlefish mantle muscle. In longfin squid mantle muscle, isometric stress was much lower in paralarvae than in adults (119 kN m^−2^ in paralarvae; 290 kN m^−2^ in adults) and appeared to be partly explained by the shorter thick filament lengths in paralarvae, compared with those of adults (Thompson et al., 2010). It is unknown whether juvenile cuttlefish have shorter thick filaments than adults; if they do, any differences were insufficient to yield significant differences in stress in juveniles compared with adults.

The absence of a plateau in force during isometric tetanic contractions (as is observed in vertebrate striated muscle) appears to be a general characteristic of molluscan muscle as the mantle muscle of European common squid (*Alloteuthis subulata*; Milligan et al., 1997), longfin squid (*D. pealeii*; Thompson et al., 2010, 2008) and European common cuttlefish (*S. officinalis*; Milligan et al., 1997), and the adductor muscle of sea scallops (*Placopecten magellanicus*; Rall, 1981) and bay scallops (*Argopecten irradians*; Olson and Marsh, 1993) all showed similar behaviour. It has been suggested that the inability to maintain force during a tetanic contraction is due to neuromuscular fatigue and reflects the non-physiological behaviour during this type of contraction in a muscle that is designed to generate mechanical work (Rall, 1981). The twitch:tetanus ratio (0.62–0.75) in cuttlefish mantle muscle fell within the range reported in other molluscs (0.10–0.89), which is typically lower than that reported in vertebrate fast-twitch muscle (e.g. 0.11–0.12 in mouse hindlimb muscles; Askew and Marsh, 1997). We suggest the higher twitch:tetanus ratio in cuttlefish may be explained by a higher proportion of the stored calcium being released during a twitch than seen in vertebrate musculature. Nesher et al. (2019) noted action potentials in octopus (*Octopus vulgaris*) arm muscle are mediated by calcium, suggesting direct activation of cephalopod musculature, which may explain higher twitch:tetanus ratios. Different excitation–contraction mechanisms have been noted in tentacle musculature; however, this muscle does not show the same oblique striation noted in mantle and arm musculature (Gilly et al., 2020).

Although the twitch kinetics differed between juvenile and adult mantle muscle, the maximum velocity of shortening was similar (3–3.2 *L* s^−1^). Differences in the maximum shortening velocity of centrally located, mitochondria-poor mantle muscle fibres have been reported in paralarval and adult longfin squid (Thompson et al., 2010, 2008). The size difference between paralarvae and adult squid is much larger than that between juvenile and adult cuttlefish, and a greater difference in *V*_max_ might therefore be expected in the squid data; however, in some systems there is a poor correlation between *V*_max_ and muscle operating frequency (Marsh, 1990). The mean shortening velocity of cuttlefish mantle muscle (3–3.2 *L* s^−1^) is similar to that of the European common squid (*A. subulata*; Milligan et al., 1997). The maximum shortening velocity in the functionally related obliquely striated funnel muscle of longfin squid is similar (2.2 *L* s^−1^; Rosenbluth et al., 2010) to that in adult cuttlefish (3 *L* s^−1^; this study). The mechanical function of these muscles is rather different: the funnel muscles play a key role in jet ejection, adjusting aperture size and amending the direction in which a jet will be ejected, while the mantle muscles power jet propulsion. However, both muscles are obliquely striated and both are involved in swimming and probably contract at similar frequencies, and therefore the similarity in their mechanical properties is unsurprising. The maximum shortening velocity of the squid tentacle muscles is substantially higher than that of the mantle muscle (approximately 15.4 *L* s^−1^ at 19°C; Kier and Curtin, 2002). Despite the higher temperature at which the measurements were made, part of the difference in *V*_max_ is due to differences in the muscle structure (tentacle muscle is cross-striated and has shorter thick filaments; Kier and Curtin, 2002) and reflects the adaptation of tentacle muscle to the role the tentacles play in prey strike. In contrast, the *V*_max_ of the squid arm muscles (also obliquely striated) is much lower (1.5 *L* s^−1^ at 19°C; Kier and Curtin, 2002) than that of the mantle muscle, and probably reflects the different mechanical role of this muscle, which is involved in relatively slow movements of the arms during swimming, prey handling and behavioural displays (Kier and Curtin, 2002). Beyond cephalopod lineages, but still within Mollusca, shortening velocities of the adductor muscle of bay scallops (*A. irradians*) is similar though a little higher than that of cuttlefish mantle muscle (5.4 *L* s^−1^ at 10°C; Olson and Marsh, 1993). The slightly higher *V*_max_ in scallops could be related to the slightly higher cycle frequencies during locomotion in scallops (Marsh and Olson, 1994) compared withs cuttlefish.

### Performance of obliquely striated muscle under cyclical contractions

During locomotion, the muscles of molluscs undergo cyclical length changes during which they are alternately actively shortened and passively lengthened in order to generate net mechanical work and power; cephalopods, gastropods and bivalves are all known to locomote in this manner (Bartol et al., 2008; Denny and Miller, 2006; Denny, 1981). The work-loop technique allows muscle performance to be investigated *in vitro* during cyclical contractions, simulating *in vivo* behaviour; however, studies to date have been restricted to bivalve molluscs, such as scallops (Marsh and Olson, 1994).

Here, we found the net power output of muscle showed a typical inverted U-shaped relationship with cycle frequency, with an optimum intermediate cycle frequency at which power was maximal. Similar relationships have been widely reported in muscles including the flight muscle of tobacco hawkmoths (*Manduca sexta*; Stevenson and Josephson, 1990), the hindlimb muscles of mice (*Mus musculus*; Askew and Marsh, 1997), the iliofibularis of desert iguanas (*Dipsosaurus dorsalis*; Swoap et al., 1993) and the white myotomal muscle of dogfishes (*Scyliorhinus canicula*; Curtin and Woledge, 1993), and is a general feature of all muscles. The nature of the relationship between power and cycle frequency is the result of the decrease in the net work generated as cycle frequency increases, resulting from the decrease in force due to both force–velocity effects and a reduction in the shortening duration, which limits the time available for muscle activation and relaxation; power is the product of work and cycle frequency. However, in both juvenile and adult cuttlefish mantle muscle, net work also exhibited an inverted U-shaped relationship with a decrease in net work at the lowest cycle frequencies. It is possible that at the lowest cycle frequencies, force was reduced as a result of fatigue, as appears to also occur during an isometric tetanic contraction where a plateau is not attained. Both juvenile and adult muscles showed this inverted U-shaped net work/power–cycle frequency relationship; however, the relationship was left-shifted to lower cycle frequencies and with a lower optimum cycle frequency for maximum net work and net power in adults compared with juveniles. This ontogenetic decrease in the optimal cycle frequency for maximum net power output has previously been widely reported in the striated muscle of both vertebrates and invertebrates (Altringham and Johnston, 1990; Altringham and Young, 1991; Perry et al., 2009; Van Wassenbergh et al., 2007). The lower optimal cycle frequency of adult mantle muscle is consistent with the slower twitch kinetics, compared with juveniles: with slower twitch kinetics, a longer period of time is required for contraction and relaxation, resulting in a lower optimal cycle frequency.

### *In vivo* swimming performance

A range of cycle frequencies (0.6–2 Hz) was used during the *in vitro* work-loop experiments, with a mean strain of 0.15 (strain amplitude ±0.075) as this was found to yield maximum net power during preliminary measurements. In juveniles, the mean cycle frequency of jetting events was 1.39 Hz (range 0.70–2.92 Hz), which corresponded to the cycle frequency found to yield the maximum net power output *in vitro.* The *in vivo* strain was 0.19 (range 0.15–0.31). During preliminary testing, a strain of 0.2 (strain amplitude ±0.1) was noted to return marginally lower net power output – approximately 15% lower than a strain of 0.15. These data suggest that juveniles are operating under conditions that maximise net muscular power output during jet propulsion swimming. As muscle was taken from the central mitochondria poor zone, the muscle is unlikely to play a role in respiration or routine swimming, being used exclusively for escape responses. Whilst we did not measure the jetting frequency in adults, the observations in juveniles suggests that the optimal frequency for net power *in vitro* is likely to correspond to the jetting frequency during swimming. Marsh and Olson (1994) also reported that the jet propulsion frequencies during swimming in bay scallops corresponded to those found to yield maximum net power output of the adductor muscle during simulated natural cycles and sinusoidal cycles *in vitro* (Marsh and Olson, 1994; Marsh et al., 1992). The maximisation of net muscle power output during free-swimming is probably of key importance, maximising energy transfer to the wake of the animal and maximising swimming performance during escape responses. Askew and Marsh (2002) noted power-generating muscles would have high mean stresses during cyclic activities, as seen here. Maximising power ensures rapid escape responses can be achieved, where such responses are of key importance when camouflage/crypsis is broken by predators.

## Supplementary Material

10.1242/jexbio.244977_sup1 Supplementary information

## References

[JEB244977C1] Altringham, J. D. and Johnston, I. A. (1990). Scaling effects on muscle function - power output of isolated fish muscle-fibers performing oscillatory work. *J. Exp. Biol.* 151, 453-467. 10.1242/jeb.151.1.453 1919410

[JEB244977C2] Altringham, J. D. and Young, I. S. (1991). Power output and the frequency of oscillatory work in mammalian diaphragm muscle - the effects of animal size. *J. Exp. Biol.* 157, 381-389. 10.1242/jeb.157.1.381 2061706

[JEB244977C3] Altringham, J. D., Morris, T., James, R. S. and Smith, C. I. (1996). Scaling effects on muscle function in fast and slow muscles of *Xenopus laevis*. *Exp. Biol.* 1, 1-8. 10.1007/s00898-996-0006-z

[JEB244977C4] Askew, G. N. and Marsh, R. L. (1997). The effects of length trajectory on the mechanical power output of mouse skeletal muscles. *J. Exp. Biol.* 200, 3119-3131. 10.1242/jeb.200.24.3119 9364020

[JEB244977C5] Askew, G. N. and Marsh, R. L. (2001). The mechanical power output of the pectoralis muscle of blue-breasted quail (*Coturnix chinensis*): the in vivo length cycle and its implications for muscle performance. *J. Exp. Biol.* 204, 3587-3600. 10.1242/jeb.204.21.3587 11719526

[JEB244977C47] Askew, G. N. and Marsh, R. L. (2002). Muscle designed for maximum short-term power output. *J. Exp. Biol.* 205, 2153-2160. 10.1242/jeb.205.15.2153 12110648

[JEB244977C6] Bartol, I. K., Patterson, M. R. and Mann, R. (2001). Swimming mechanics and behavior of the shallow-water brief squid *Lolliguncula brevis*. *J. Exp. Biol.* 204, 3655-3682. 10.1242/jeb.204.21.3655 11719531

[JEB244977C7] Bartol, I. K., Krueger, P. S., Thompson, J. T. and Stewart, W. J. (2008). Swimming dynamics and propulsive efficiency of squids throughout ontogeny. *Integr. Comp. Biol.* 48, 720-733. 10.1093/icb/icn043 21669828

[JEB244977C8] Bouchaud, O. (1991). Energy consumption of the cuttlefish *Sepia officinalis* L. (Mollusca, Cephalopoda) during embryonic development, preliminary-results*.* *Bull. Mar. Sci.* 49, 333-340.

[JEB244977C9] Caiozzo, V. J. (2002). Plasticity of skeletal muscle phenotype: Mechanical consequences. *Muscle Nerve* 26, 740-768. 10.1002/mus.10271 12451599

[JEB244977C10] Curtin, N. A. and Woledge, R. C. (1993). Efficiency of energy-conversion during sinusoidal movement of white muscle-fibers from the dogfish *Scyliorhinus canicula*. *J. Exp. Biol.* 183, 137-147. 10.1242/jeb.183.1.137 1919411

[JEB244977C11] Curtin, N. A., Woledge, R. C. and Bone, Q. (2000). Energy storage by passive elastic structures in the mantle of *Sepia officinalis*. *J. Exp. Biol.* 203, 869-878. 10.1242/jeb.203.5.869 10667969

[JEB244977C12] Denny, M. W. (1981). A quantitative model for the adhesive locomotion of the terrestrial slug, *Ariolimax columbianus*. *J. Exp. Biol.* 91, 195-217. 10.1242/jeb.91.1.195

[JEB244977C13] Denny, M. and Miller, L. (2006). Jet propulsion in the cold: mechanics of swimming in the Antarctic scallop *Adamussium colbecki*. *J. Exp. Biol.* 209, 4503-4514. 10.1242/jeb.02538 17079720

[JEB244977C14] Gilly, W. F., Renken, C., Rosenthal, J. J. C. and Kier, W. M. (2020). Specialization for rapid excitation in fast squid tentacle muscle involves action potentials absent in slow arm muscle. *J. Exp. Biol.* 223, jeb218081. 10.1242/jeb.218081 31900349

[JEB244977C15] Girgenrath, M. and Marsh, R. L. (1997). In vivo performance of trunk muscles in tree frogs during calling. *J. Exp. Biol.* 200, 3101-3108. 10.1242/jeb.200.24.3101 9364018

[JEB244977C16] Gladman, N. W. (2018). The energetics and mechanics of jet propulsion swimming in European common cuttlefish (*Sepia officinalis*). *PhD thesis*, University of Leeds. https://etheses.whiterose.ac.uk/22890/10.1242/jeb.246225 PMC10560557 37655637

[JEB244977C17] James, R. S., Cole, N. J., Davies, M. L. F. and Johnston, I. A. (1998). Scaling of intrinsic contractile properties and myofibrillar protein composition of fast muscle in the fish *Myoxocephalus scorpius* L. *J. Exp. Biol.* 201, 901-912. 10.1242/jeb.201.7.901 9487095

[JEB244977C18] Johnson, T. P., Swoap, S. J., Bennett, A. F. and Josephson, R. K. (1993). Body size, muscle power output and limitations on burst locomotor performance in the lizard *Dipsosaurus dorsalis*. *J. Exp. Biol.* 174, 199-213. 10.1242/jeb.174.1.199

[JEB244977C48] Josephson, R. K. (1985). Mechanical power output from striated muscle during cyclic contraction. *J. Exp. Biol.* 114, 493-512. 10.1242/jeb.114.1.493

[JEB244977C19] Josephson, R. K. (1993). Contraction dynamics and power output of skeletal muscle. *Annu. Rev. Physiol.* 55, 527-546. 10.1146/annurev.ph.55.030193.002523 8466183

[JEB244977C20] Kier, W. M. and Curtin, N. A. (2002). Fast muscle in squid (*Loligo pealei*): contractile properties of a specialized muscle fibre type. *J. Exp. Biol.* 205, 1907-1916. 10.1242/jeb.205.13.1907 12077167

[JEB244977C21] Kier, W. M. and Schachat, F. H. (2008). Muscle specialization in the squid motor system. *J. Exp. Biol.* 211, 164-169. 10.1242/jeb.008144 18165243

[JEB244977C22] Kier, W. M. and Thompson, J. T. (2003). Muscle arrangement, function and specialization in recent coleoids. *Berliner Palaobiol. Abh.* 3, 141-162.

[JEB244977C23] Lamarre, S. G., Maccormack, T. J., Bourloutski, E., Callaghan, N. I., Pinto, V. D., Andrade, J. P., Sykes, A. V. and Driedzic, W. R. (2019). Interrelationship between contractility, protein synthesis and metabolism in mantle of juvenile cuttlefish (*Sepia officinalis*). *Front. Physiol.* 10, 1051. 10.3389/fphys.2019.01051 31507433 PMC6716058

[JEB244977C24] Marsh, R. L. (1988). Ontogenesis of contractile properties of skeletal-muscle and sprint performance in the lizard *Dipsosaurus dorsalis*. *J. Exp. Biol.* 137, 119-139. 10.1242/jeb.137.1.119 3209964

[JEB244977C25] Marsh, R. L. (1990). Deactivation rate and shortening velocity as determinants of contractile frequency. *Am. J. Physiol.* 259, R223-R230. 10.1152/ajpregu.1990.259.2.R223 2201216

[JEB244977C26] Marsh, R. L. and Bennett, A. F. (1986). Thermal-dependence of contractile properties of skeletal-muscle from the lizard *Sceloporus occidentalis* with comments on methods for fitting and comparing force-velocity curves. *J. Exp. Biol.* 126, 63-77. 10.1242/jeb.126.1.63 3806003

[JEB244977C27] Marsh, R. L. and Olson, J. M. (1994). Power output of scallop adductor muscle during contractions replicating the *in-vivo* mechanical cycle. *J. Exp. Biol.* 193, 139-156. 10.1242/jeb.193.1.139 7964397

[JEB244977C28] Marsh, R. L., Olson, J. M. and Guzik, S. K. (1992). Mechanical performance of scallop adductor muscle during swimming. *Nature* 357, 411-413. 10.1038/357411a0 1594046

[JEB244977C29] Mendez, J. and Keys, A. (1960). Density and composition of mammalian muscle. *Meta. Clin. Exp.* 9, 184-188.

[JEB244977C30] Milligan, B. J., Curtin, N. A. and Bone, Q. (1997). Contractile properties of obliquely striated muscle from the mantle of squid (*Alloteuthis subulata*) and cuttlefish (*Sepia officinalis*). *J. Exp. Biol.* 200, 2425-2436. 10.1242/jeb.200.18.2425 9320349

[JEB244977C31] Nesher, N., Maiole, F., Shomrat, T., Hochner, B. and Zullo, L. (2019). From synaptic input to muscle contraction: arm muscle cells of Octopus vulgaris show unique neuromuscular junction and excitation-contraction coupling properties. *Proc. R. Soc. B Biol. Sci.* 286, 20191278. 10.1098/rspb.2019.1278 PMC6732383 31455193

[JEB244977C32] Olson, J. M. and Marsh, R. L. (1993). Contractile properties of the striated adductor muscle in the bay scallop *Argopecten irradians* at several temperatures. *J. Exp. Biol.* 176, 175-193. 10.1242/jeb.176.1.175 8478601

[JEB244977C33] Perry, M. J., Tait, J., Hu, J., White, S. C. and Medler, S. (2009). Skeletal muscle fiber types in the ghost crab, *Ocypode quadrata*: implications for running performance. *J. Exp. Biol.* 212, 673-683. 10.1242/jeb.023481 19218519

[JEB244977C34] Rall, J. A. (1981). Mechanics and energetics of contraction in striated-muscle of the sea scallop, *Placopecten magellanicus*. *J. Physiol.* 321, 287-295. 10.1113/jphysiol.1981.sp013984 6978395 PMC1249626

[JEB244977C35] Rogers, C. M., Nelson, L., Milligan, B. J. and Brown, E. R. (1997). Different excitation-contraction coupling mechanisms exist in squid, cuttlefish and octopod mantle muscle. *J. Exp. Biol.* 200, 3033-3041. 10.1242/jeb.200.23.3033 9359892

[JEB244977C36] Rosenbluth, J., Szent-Gyorgyi, A. G. and Thompson, J. T. (2010). The ultrastructure and contractile properties of a fast-acting, obliquely striated, myosin-regulated muscle: the funnel retractor of squids. *J. Exp. Biol.* 213, 2430-2443. 10.1242/jeb.037820 20581273 PMC2892422

[JEB244977C37] Schneider, C. A., Rasband, W. S. and Eliceiri, K. W. (2012). NIH Image to ImageJ: 25 years of image analysis. *Nat. Methods* 9, 671-675. 10.1038/nmeth.2089 22930834 PMC5554542

[JEB244977C38] Shaffer, J. F. and Kier, W. M. (2012). Muscular tissues of the squid *Doryteuthis pealeii* express identical myosin heavy chain isoforms: an alternative mechanism for tuning contractile speed. *J. Exp. Biol.* 215, 239-246. 10.1242/jeb.064055 22189767 PMC3244340

[JEB244977C39] Stevenson, R. D. and Josephson, R. K. (1990). Effects of operating frequency and temperature on mechanical power output from moth flight-muscle. *J. Exp. Biol.* 149, 61-78. 10.1242/jeb.149.1.61

[JEB244977C40] Swoap, S. J., Johnson, T. P., Josephson, R. K. and Bennett, A. F. (1993). Temperature, muscle power output and limitations on burst locomotor performance of the lizard *Dipsosaurus dorsalis*. *J. Exp. Biol.* 174, 185-197. 10.1242/jeb.174.1.185

[JEB244977C41] Thompson, J. T. and Kier, W. M. (2006). Ontogeny of mantle musculature and implications for jet locomotion in oval squid *Sepioteuthis lessoniana*. *J. Exp. Biol.* 209, 433-443. 10.1242/jeb.02017 16424093

[JEB244977C42] Thompson, J. T., Szczepanski, J. A. and Brody, J. (2008). Mechanical specialization of the obliquely striated circular mantle muscle fibres of the long-finned squid *Doryteuthis pealeii*. *J. Exp. Biol.* 211, 1463-1474. 10.1242/jeb.017160 18424680

[JEB244977C43] Thompson, J. T., Bartol, I. K., Baksi, A. E., Li, K. Y. and Krueger, P. S. (2010). The ontogeny of muscle structure and locomotory function in the long-finned squid *Doryteuthis pealeii*. *J. Exp. Biol.* 213, 1079-1091. 10.1242/jeb.034553 20228344

[JEB244977C49] Thompson, J. T., Shelton, R. M. and Kier, W. M. (2014). The length-force behavior and operating length range of squid muscle vary as a function of position in the mantle wall. *J. Exp. Biol.* 217, 2181-2192. 10.1242/jeb.083907 24675565

[JEB244977C44] Van Wassenbergh, S., Herrel, A., James, R. S. and Aerts, P. (2007). Scaling of contractile properties of catfish feeding muscles. *J. Exp. Biol.* 210, 1183-1193. 10.1242/jeb.000109 17371917

[JEB244977C45] Ward, D. V. and Wainwright, S. A. (1972). Locomotory aspects of squid mantle structure. *J. Zool.* 167, 437-449. 10.1111/j.1469-7998.1972.tb01735.x

[JEB244977C46] Wells, M. J. (1995). The evolution of a racing snail. *Mar. Freshw. Behav. Physiol.* 25, 1-12. 10.1080/10236249409378904

