# Supplementary material for: The mechanical properties of the mantle muscle of European cuttlefish (Sepia officinalis)
Source: J Exp Biol. 2022 Dec 15;225(23):jeb244977. doi: 10.1242/jeb.244977 (PMC10112868; doi:10.1242/jeb.244977)
Supplement: Supplementary information [file jexbio-225-244977-s1.pdf]

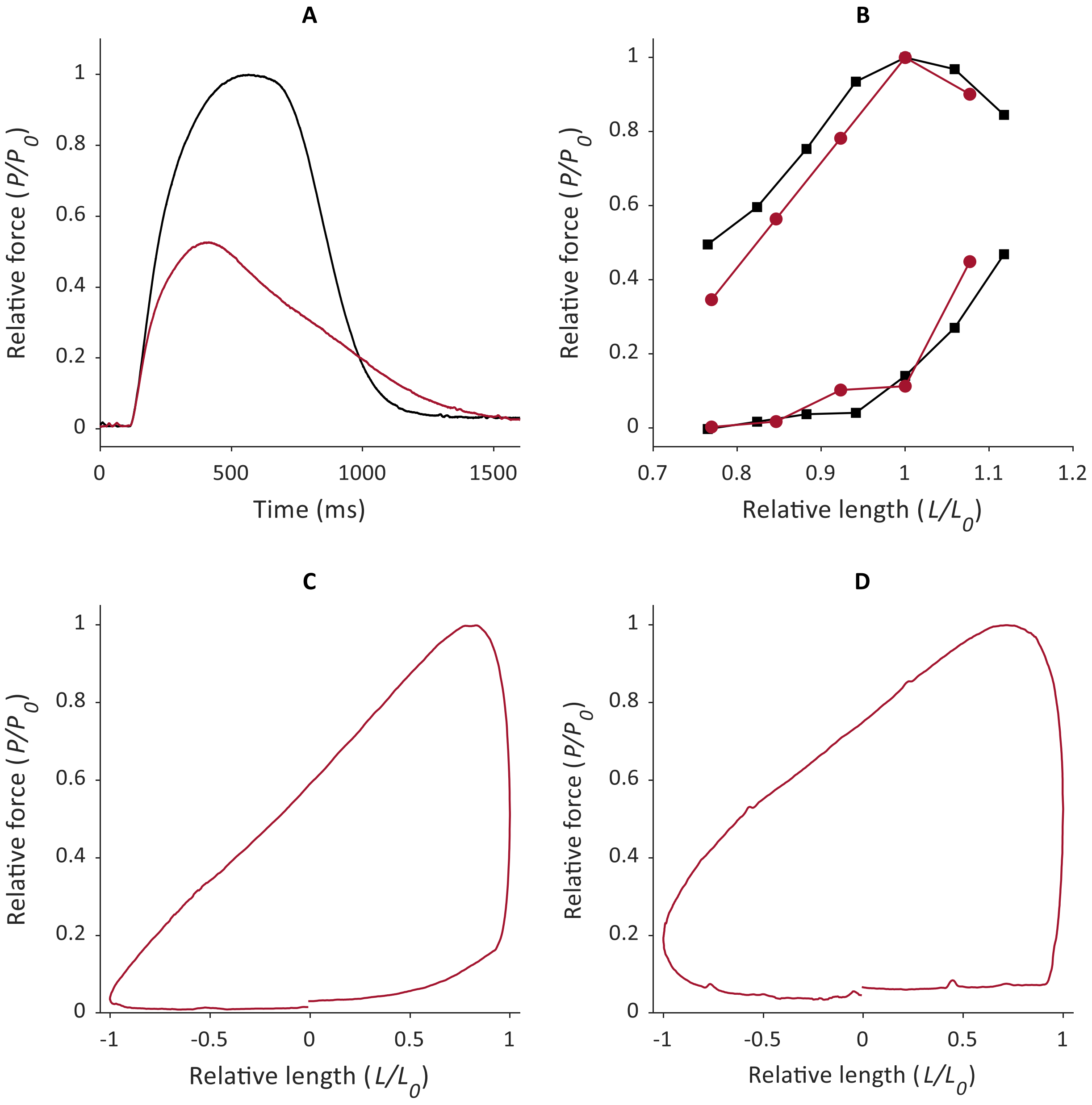

Fig. S1. Example isometric and cyclic muscle properties of cuttlefish mantle muscle. (A) Example force output of a single twitch (red) and tetanus (black) from one adult cuttlefish preparation. Force is normalised to the peak tetanic force as P/P_0. (B) Example active and passive force-length relationships of one adult (black) and juvenile (red) preparation. All values expressed relative to L_0 and P_0. (C) Example juvenile work loop at 0.8 Hz and (D) example adult work loop at 0.8 Hz

Table S1. Details of stimulus train durations used to elicit peak power output of cuttlefish muscle

**Cycle frequency (Hz)**	**Train duration (ms)**
**0.6**	800
**0.8**	475
**1.0**	350
**1.2**	260
**1.4**	200
**1.6**	85
**1.8**	72
**2.0**	65

Journal of Experimental Biology • Supplementary information
